# Identification of conserved genes triggering puberty in European sea bass males (*Dicentrarchus labrax*) by microarray expression profiling

**DOI:** 10.1186/s12864-017-3823-2

**Published:** 2017-06-05

**Authors:** Mercedes Blázquez, Paula Medina, Berta Crespo, Ana Gómez, Silvia Zanuy

**Affiliations:** 1 Instituto de Acuicultura de Torre la Sal, Consejo Superior de Investigaciones Científicas (IATS-CSIC), Ribera de Cabanes, 12595 Castellón, Spain; 2 Instituto de Ciencias del Mar, Consejo Superior de Investigaciones Científicas (ICM-CSIC), Passeig Maritim 37-49, 08003 Barcelona, Spain; 3 Present address: Universidad de Antofagasta, Avda Angamos 601, Antofagasta, Chile; 4 Present address: UCL Great Ormond Street Institute of Child Health, 30 Guilford Street, London, WC1N 1EH UK

**Keywords:** Spermatogenesis, Cell cycle, Meiosis, Retinoic acid, Teleosts

## Abstract

**Background:**

Spermatogenesis is a complex process characterized by the activation and/or repression of a number of genes in a spatio-temporal manner. Pubertal development in males starts with the onset of the first spermatogenesis and implies the division of primary spermatogonia and their subsequent entry into meiosis. This study is aimed at the characterization of genes involved in the onset of puberty in European sea bass, and constitutes the first transcriptomic approach focused on meiosis in this species.

**Results:**

European sea bass testes collected at the onset of puberty (first successful reproduction) were grouped in stage I (resting stage), and stage II (proliferative stage). Transition from stage I to stage II was marked by an increase of 11ketotestosterone (11KT), the main fish androgen, whereas the transcriptomic study resulted in 315 genes differentially expressed between the two stages. The onset of puberty induced 1) an up-regulation of genes involved in cell proliferation, cell cycle and meiosis progression, 2) changes in genes related with reproduction and growth, and 3) a down-regulation of genes included in the retinoic acid (RA) signalling pathway. The analysis of GO-terms and biological pathways showed that cell cycle, cell division, cellular metabolic processes, and reproduction were affected, consistent with the early events that occur during the onset of puberty. Furthermore, changes in the expression of three RA nuclear receptors point at the importance of the RA-signalling pathway during this period, in agreement with its role in meiosis.

**Conclusion:**

The results contribute to boost our knowledge of the early molecular and endocrine events that trigger pubertal development and the onset of spermatogenesis in fish. These include an increase in 11KT plasma levels and changes in the expression of several genes involved in cell proliferation, cell cycle progression, meiosis or RA-signalling pathway. Moreover, the results can be applied to study meiosis in this economically important fish species for Mediterranean countries, and may help to develop tools for its sustainable aquaculture.

**Electronic supplementary material:**

The online version of this article (doi:10.1186/s12864-017-3823-2) contains supplementary material, which is available to authorized users.

## Background

Puberty in fish, as in other vertebrates, comprises the developmental process during which an immature individual acquires for the first time the ability to undergo sexual reproduction [1, 2]. In teleost males, puberty is tightly regulated and implies the proliferation and division of spermatogonia (mitotic phase), their subsequent entry into meiosis with the appearance of spermatocytes (meiotic phase), and the final formation of the spermatids and the haploid mature spermatozoa [2, 3]. A species-specific number of genetically determined divisions characterize the mitotic phase [3], whereas the meiotic phase remains under the influence of the retinoic acid (RA) signalling pathway [4, 5]. Somatic Sertoli cells are important players during spermatogenesis, exhibiting a high mitotic activity, particularly at the beginning of each seasonal cycle [2], and are essential for the proliferation and differentiation of germ cells [6]. Mitosis and meiosis reveal thus as key processes for the onset of puberty in vertebrates. Meiosis is of particular importance since it implies the recombination and reduction of the genetic material, essential to ensure the correct formation of gametes, and therefore guarantees the reproduction and maintenance of the species.

Spermatogenesis is marked by the functional stimulation of the brain-pituitary-gonad (BPG) axis, responsible for its neuroendocrine control [7]. The brain is the central organ that integrates the circuits that sense the internal and external stimuli and secretes different neuropeptides that control the production of gonadotropins from the pituitary. It is generally accepted that gonadotropins (follicle stimulating hormone; Fsh and luteinizing hormone; Lh), and androgens are the main internal stimuli for vertebrate spermatogenesis. Both gonadotropins become activated by a number of factors among which the metabolic status of the individual, in terms of body size and visceral fat content, or the photoperiod, are worth mentioning [1, 2, 8, 9]. 11ketotestosterone (11KT) is the main androgen in fish and plays an important role in the progression of spermatogenesis [10]. It is involved in the proliferation of spermatogonia towards meiosis [11] and mediates the action of several factors produced by Sertoli cells like antimüllerian hormone (Amh) and insulin-like growth factors (Igfs) at the start of the cycle [12]. In addition, Fsh has been shown to stimulate 11KT production in several fish species [13–17]. In fact, Fsh receptor is present not only in Sertoli cells but also in Leydig cells, the somatic cells with steroidogenic capability, as shown in Senegalese sole [13] African catfish [14] and zebrafish [15–17].

The European sea bass (*Dicentrarchus labrax*) is an important fish species for marine aquaculture that after intense research on its reproductive function has become a model for both basic and applied research. A number of studies focused on the endocrine control of reproduction shed light on the process and aided to develop protocols for its control in captivity (reviewed by [1]). Increased growth rates under intensive culture resulted in precocious puberty in about 20–30% of males by the end of the first year of life [18], something that in normal conditions occurs during the second year [19]. As in other farmed fish, precocious maturation is one of the main drawbacks for its culture [9], resulting in important economic losses since by the time of marketing during the second year, precocious males exhibit a smaller size than that of normal-maturing males [20, 21]. Several studies have shown that this problem can be partially solved by a well-planned strategy of photoperiod control [18, 21–23], although clearly, more work is needed to understand why males mature precociously. Brain factors including kisspeptins [24–27], gonadotropin-releasing hormones (Gnrhs) [28, 29], leptin and their receptors [8] have been characterized in this species. Regarding pituitary gonadotropins, both Fsh and Lh receptors have been cloned [30], and different assays are currently available to measure Fsh and Lh levels in plasma and pituitary [31–33]. Moreover, recombinant European sea bass gonadotropins have been used as a biotechnological approach in gene therapy for assisted reproduction [34, 35]. In addition, secretion patterns of sex steroids [36], sex steroid receptors, and several steroidogenic enzymes [30, 37] during the seasonal cycle have also been reported.

Despite all the previous knowledge, little is known about the molecular machinery that triggers puberty in European sea bass males, apart from a study reporting the possible involvement of several genes in the early events preceding gonadal maturation [38]. The recent availability of several molecular resources, including a partially annotated European sea bass genome database [39] gave us the opportunity to study this process using a high throughput strategy. The aim of the present work is to compare the transcriptome from European sea bass testes before and right at the start of puberty, and to identify potential genes and pathways involved in the process. This will boost our knowledge of the onset of pubertal development from a basic perspective and will help to implement tools for the improvement of a sustainable aquaculture.

## Methods

### Animals, rearing conditions and samplings

European sea bass hatched in April 2008 at the Ecloserie Marine de Gravelines (EMG) in the North of France and were grown there until 100 days post hatching (dph) when they were transported to our aquaria facilities at the Institute of Aquaculture Torre la Sal (IATS), a research centre belonging to the Spanish National Research Council (CSIC) in the Spanish Mediterranean coast. The facilities were approved for animal experimentation by the Ministry of Agriculture and Fisheries and by the Department of Fisheries from the Generalitat Valenciana (certificate number ES120330001055). Fish were reared in 2000 l round fiberglass tanks under natural conditions of photoperiod and temperature until the end of the experiment. In December 2008, coinciding with the first breeding season in this species, fish were subjected to abdominal massage to check for the presence of sperm. This allowed us to eliminate males exhibiting precocious puberty that could interfere with our results. These procedures were repeated every 20 days for a period of about 3 months. Prior to the start of the second breeding season (August 2009), coinciding with European sea bass normal puberty, a sampling procedure was designed to obtain testes covering the first stages of spermatogenesis. Samplings (15 fish per sampling point) were performed every 10 days starting in mid-August and finishing by the end of October. At each sampling point, fish were anesthetized with 2-phenoxyethanol (0.2 ml·l^−1^) and blood was taken from the caudal vein for plasma sex-steroid measurements. Fish were subsequently sacrificed by quickly severing their spinal cord and gonads were dissected for histology (the central part of the gonad) whereas the rest was kept at −80 °C for further RNA extractions needed for the different analyses including microarray hybridizations, validations, and tissue specific expression studies. Fish were treated in agreement with the Spanish regulations (Royal Decree Act 53/2013) and the European legislation (2010/63 EU) concerning the protection of animals used for experimental and other scientific purposes. All steps were taken to reduce suffering of the animals.

### Histological analysis

After dissection, the central part of the testes was immediately fixed in 4% formaldehyde: 1% glutaraldehyde in phosphate buffered saline (PBS; [40]). Tissues were washed in PBS and dehydrated in an increasing series of ethanol 70–96%. Samples were embedded in glycol methacrilate resin (Technovit 7100; Heraeus, Kulzer, Germany), sectioned at 3–4 μm, and stained as in [41]. The stages of testicular development and the type and abundance of germ cells in each stage were assessed according to [18] and [42], respectively.

### Steroid analysis by enzyme immune assay (EIA)

Plasma levels of 11KT were determined by enzyme immune assay (EIA) in 20 fish selected from each developmental stage, using the protocol by [22]. Briefly, antibodies were used at a final concentration of 1:200,000 and the tracer (Cayman chemicals, MI, USA) was diluted at 1:50 Ellman Units (UE)/ml (used at 0.1042 EU/ml). The sensitivity of the assay was around 0.003 ng/ml (Bi/B0 = 90%) and half displacement (Bi/B0 = 50%) occurred around 0.03 ng/ml (slope = −1.018). The inter-assay coefficient of variation (*n* = 2 plates) was 1.72%.

### RNA isolation and cDNA synthesis

For hybridizations and real-time validations, testes (approx. 50–100 mg) were homogenized in Trizol (Invitrogen, Carlsbad, CA) using the FastPrep® Instrument (Qbiogene, Inc., Carlsbad, CA), a tissue homogenizer with ceramic spheres as a lysing matrix. Total RNA was extracted from the lysate with the PureLink™ RNA mini Kit (Invitrogen), following the manufacturer’s instructions. Briefly, RNA was phase separated, washed, and finally eluted in DEPC water. For the tissue-distribution study, tissues including telencephalon, hypothalamus, cerebellum, spleen, gills, head kidney, kidney, liver, testis, ovary, heart and gut were homogenized in a thioglycerol-based buffer included in the Maxwell® 16 LEV simplyRNA tissue kit (Promega, Madison, WI). The homogenates were used for RNA isolation with the Maxwell® 16 instrument (Promega) following the manufacturer’s instructions that include a DNase treatment. Nevertheless, an additional test was done on the RNAs to discard any possible DNA contamination. For microarray hybridizations RNA quality was assessed with a Bioanalyzer 2100 (RNA 6000 Nano LabChip kit Agilent, Spain) and only RNAs with RIN values higher than 8.5 were used. For other downstream applications such as quantitative real time PCR (qPCR) or conventional PCR (tissue expression study), RNA quantification was done with a Nanodrop 2000c (Thermo Scientific, Wilmington, DE) and stored at −80 °C until further cDNA synthesis. Total RNA (3 μg) was reverse transcribed to cDNA with Superscript III (Invitrogen) and random hexamers following the manufaturer’s instructions. Protection of RNA from ribonucleases during cDNA synthesis was done by including 40 units of RNAse inhibitors (RNasin, Promega). The reaction was inactivated at 70 °C for 15 min.

### Microarray hybridization and analysis

RNA labelling, hybridizations, and scanning were performed at the Autonomous University of Barcelona (UAB). Total RNA (100 ng) was amplified and Cy3-labeled with One-Color Microarray Gene Expression Analysis (Low Input Quick Amp Labelling kit, Agilent) along with One-Color RNA SpikeIn Kit (Agilent) following the manufacturer’s instructions. The resulting cRNA was purified (RNeasy mini spin columns; Qiagen), quantified with a Nanodrop ND-1000 and checked with a Bioanalyzer 2100 as previously described. Amplified samples (1.65 μg per sample) were hybridized to a custom oligonucleotide high-density European sea bass microarray (Agilent 4 × 44 K design format; http://www.agilent.com/) containing 60-mer oligonucleotides with a linker directly spotted on glass slides using the Agilent’s SurePrint Tecnology. Three samples from each testicular stage, selected after histological examination and 11KT plasma levels, were used for microarray hybridizations, each of them consisting of a pool RNAs from six males. The pools were used as biological replicates and thus independent samples for microarray hybridizations. In addition, and since each microarray plate can hold up to four samples (4 × 44 design), one sample from each stage was randomly chosen and hybridized in both plates as a quality control to check for possible inter-plate hybridization differences. The probes contained in the microarray (GEO accession number GPL13443) cover 13,199 unique sequences of *Dicentrarchus labrax* that include 6275 annotated transcripts, each with 3 specific probes, and 6924 ESTs with 1 probe/target sequence. Assuming that a typical diploid teleost genome is expected to have 26–28 thousand protein coding loci, the microarray used for the study should cover about half of the genes of the species. Hybridizations were done at 65 °C for 17 h (GE Hybridization Kit; Agilent). Washes were conducted as recommended by the manufacturer using Agilent’s Gene Expression Wash Pack with stabilization and drying solution and arrays were scanned with a G2505B (Agilent). Several quality control features and spot intensities were extracted with Agilent’s Feature Extraction software v10.4. Finally, data were analyzed with GeneSpring software v10.1. Percentile shift normalization was used to adjust all spot intensities in the array (percentile target = 75). Principal Component Analysis (PCA) was used as a quality control on samples and allowed to decrease the number of false positives before the statistical analysis. Normalized data were filtered by comparison of the standard deviation expression among groups (filter by expression). Statistical analyses were performed on filtered data using a t-test. Significant differences in the transcriptomic profile between early stages of spermatogenesis (data filtered at a fold change (FC) expression of 2) were set at *p* < 0.01. The corresponding study was deposited at the Gene Expression Omnibus (GEO-NCBI) database under the accession number GSE47400.

### Gene annotation and enrichment analysis

The web-based tools Genecards (http://www.genecards.org), Uniprot (http://www.uniprot.org) and AmiGO 2 (amigo.http://amigo.geneontology.org) were used to assign gene names, synonyms and functions to the differentially expressed genes (DEGs) found after microarray hybridizations. The annotation of the sequences was manually curated, improving the accuracy of the information obtained from the microarray used for this study. A further improvement was added implementing the Blast2Go software [43] that enriched the number of GO-term annotations. A list containing all genes included in our custom-made microarray was used as a reference set to evaluate the enrichment in GO-terms in the subset of DEGs. The resulting data were analysed with Fisher’s exact test with multiple testing correction of the false discovery rate. In addition, annotated DEGs were ascribed to functional biological pathways using the Kyoto Encyclopaedia of Genes and Genomes (http://www.genome.jp/kegg) and the possible altered metabolic pathways were assessed.

### Array validation by quantitative real-time PCR (qPCR)

EST sequences of the DEGs were used as a query in Blast searches against the European sea bass genome and GeneBank databases in order to position the selected DEGs in their corresponding genes. Primers for the amplification of the DEGs were designed in areas covering intron-exon boundaries to check for genomic contamination using Primer 3 (http://primer3.ut.ee). Primers (Additional file 1) were checked by conventional PCR and the amplified fragments sequenced to verify their identities. qPCR analyses were performed with an iCycler iQ™ (BioRad Labs., Inc.) using SYBR® Green (PCR Master Mix; Applied Biosystems). PCR reactions were run in triplicate in optically clear 96-well plates in a final 20 μl volume containing 10 μl of 2× Sybr Green Master mix, 10 pmol of each primer and 5 μl of diluted cDNA (1:50 for the target genes or 1:500 for the reference gene). Cycling parameters included an initial denaturation at 95 °C for 3 min, followed by 40 cycles at 95 °C for 15 s and annealing-extension at 60–72 °C for 1 min ending with an extension at 72 °C for 1 min. A final temperature dissociation step was done to ensure the presence of just one product. qPCR data were collected with iCycler™ iQ optical system software (v. 3.0, BioRad). The cycle threshold (Ct) was calculated as the average of three replicates per sample. Gene expression analyses were conducted using the Q-Gene core module [44]. Briefly, for each gene the amplification efficiency (E) was calculated from the slope of the linear correlation between Cts and the logarithm of the amount of serially diluted RNA, used as a standard, following the eq. E = 10^(−1/slope)^. E values for the different genes were within the range of 93.5–101.8%. Values were normalized (normalized expression; NE) to the constitutively expressed reference gene *18S rRNA* in each sample (*n* = 8 individual fish per stage and gene) according to the eq. NE = (E_ref_)^Ctref^/(E_target_)^Cttarget^. *18S rRNA* was considered a good reference gene since it exhibited the best bestkeeper index when comparing different developmental stages [45]. In addition, the expression of this gene remains constant in many physiological conditions such as differentiation and proliferation [46] making it a suitable reference gene for this study.

### Tissue specific expression

The expression of the selected DEGs was assessed in different tissues including telencephalon, hypothalamus, cerebellum, spleen, gills, head kidney, kidney, liver, testis, ovary, heart and gut. PCR reactions were performed with an initial denaturation of 5 min at 94 °C, and then 34 cycles with the following characteristics: denaturation at 94 °C for 30 s, annealing at 60 °C for 30 s, and extension at 72 °C for 30 s. A final extension of 2 min at 72 °C was added at the end of the 34 cycles.

### Sequencing, cloning, and phylogenetic studies of European sea bass *cyp26a1*

Based on the ESTs of the microarray, the full sequence of European sea bass *cyp26a1* was localized along the genome. Specific primers were designed in 3′- and 5′-UTR flanking regions to amplify its full-coding sequence. The fragment was cloned into a bacterial vector using the pGEM T-easy cloning kit (Promega Corp., Madison, WI), and amplified in *E. coli* competent cells following the manufacturer’s instructions. Several colonies were selected, grown in liquid LB and finally sequenced with an automatic ABI 3100 Genetic Analyser (Applied Biosystems, Foster City, CA), using the BigDye Terminator v3.1 Cycle Sequencing Kit (Applied Biosystems). The identity of the clones was confirmed after sequencing, multiple alignment comparisons, and phylogenetic analysis. An in silico study of the 5′ upstream 1500 bp of the flanking promoter sequence using MatInspector and (Promo) Transfac v.8.3 was used to identify the presence of putative binding sites for transcription factors that could be involved in the activation or repression of *cyp26a1* transcription. An alignment of known Cyp26 proteins from vertebrates, either compiled from GenBank/EMBL or predicted in ENSEMBL, was made with clustalW2. For the phylogenetic tree, the distances were computed with the Poisson correction method [47] and the evolutionary history was inferred using the Neighbor-Joining method [48] after a bootstrap test using 1000 replicates. The phylogenetic analysis was carried out in MEGA v.4 [49].

### Statistical analyses

Student’s t-test for hormonal analysis, microarray hybridizations and gene expression levels was used to reveal significant differences between stage I and stage II. In all cases, significant differences were accepted at *p* < 0.05 except for microarray hybridizations for which differences were accepted at *p* < 0.01.

## Results

### Sample selection: histological and hormonal classification

**Fig. 1:**
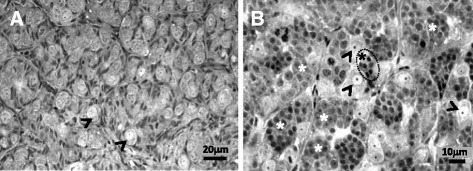
Photomicrographs of European sea bass testis during early stages of pubertal development. **a** Sexually immature testis in stage I, was characterized by the presence of type A spermatogonia (*arrowheads*) located within the seminiferous lobules (**b**) Early recrudescent testis in stage II, characterized by the presence of type A spermatogonia (*arrowheads*), abundant cysts of type B spermatogonia (*white asterisks*), and scarce cysts of type I spermatocytes (*encircled black asterisk*)

**Fig. 2:**
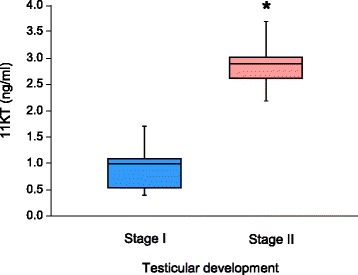
Box-and-whisker plots of 11 ketotestosterone (11KT) plasma levels in European sea bass males during early stages of puberty. 11KT levels were measured at two different stages of pubertal development: stage I, corresponding to sexually immature testes and stage II corresponding to early recrudescent testes. Box represents uper and lower quartiles and maximum and minimum observed values are represented by whiskers. Horizontal line represents the median value (0.99 ng/ml for stage I and 2.90 ng/ml for stage II). The asterisk denotes statistical differences between both groups after a student t-test (*p* < 0.05); *n* = 20 in each experimental group

Sea bass testes were histologically classified according to their stage of spermatogenesis [18, 42]. Since the study was focussed on the onset of spermatogenesis, only testes in stage I (immature) and stage II (proliferative) were used. Briefly, stage I, corresponded to an immature testis, and was characterized by the presence of type A spermatogonia located within the seminiferous lobules (Fig. 1a). Stage II, corresponded to testis in a proliferative phase and was characterized by the presence of type A spermatogonia, abundant cysts of type B spermatogonia and sometimes cysts of type I spermatocytes (Fig. 1b). Plasma samples from males previously classified by histology as stage I (*n* = 20) or stage II (*n* = 20) were used to check their levels of 11KT. The results showed that 11KT was a suitable marker to discriminate between males in stage I and stage II with significantly higher levels in the latter than the former (Fig. 2). Based on that, testes from 18 males in stage I (11KT levels ranging between 0.35 and 0.87 ng/ml), and 18 males in stage II (11KT levels ranging between 2.18 and 3.64 ng/ml) were selected for further RNA extractions and microarray hybridizations. For that purpose, the RNAs from the 18 males of each testicular stage were randomly divided and pooled into six groups, three corresponding to stage I and the other three to stage II. The pools of each stage consisted of the RNA from six different fish of that particular stage up to 18 fish per stage. The pools were used as biological replicates and thus independent samples for microarray hybridizations.

### Microarray hybridizations

**Fig. 3:**
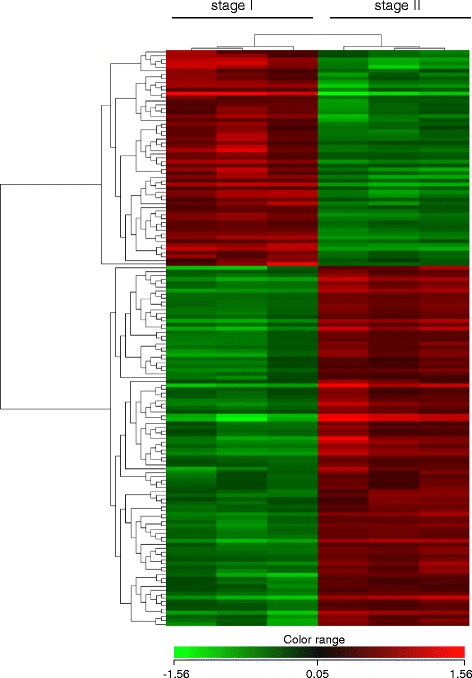
Hierarchical heat map of European sea bass annotated ESTs differentially expressed during early stages of puberty. The individual genes are pictured horizontally showing their relative expression values across all replicates of the different stages of pubertal development (tree replicates per stage) that are represented in each column. The colour scheme is calibrated to the log2 expression values with red representing higher transcript abundance and green lower transcript abundance. The heatmap displays only DEGs (corresponding to 152 annotated genes) with significantly different expression values (*p* < 0.01) between stage I and stage II and a log2 fold change value greater than two (Pearson correlation). The scale bar shows Z-score values

Changes in gene expression during the onset of spermatogenesis (stage I versus stage II) were assessed with a European sea bass specific microarray previously described and validated (GPL13443). The study identified 315 DEGs between the two spermatogenic stages (FC > 2), among which 162 corresponded to functionally annotated genes whereas the remaining 153 were non-annotated sequences. When comparing their expression, a similar number of DEGs were found to be upregulated and downregulated (150 upregulated versus 165 downregulated; see Additional file 2 for a list of all DEGs and Additional file 3 for a glossary of the genes involved in cell proliferation, reproduction, growth and RA-signalling pathway with particular mention in this study). A PCA showed the spatial distribution of the microarray data and revealed the presence of two clear clusters, one corresponding to stage I testes and the other one to stage II testes (Additional file 4). Component 1 explained 92.43% of the variation whereas component 2 was responsible for 5.13% of the variation. In addition, a heatmap representation of the DEGs grouped fish according to their stage of testicular development (Fig. 3).

A Gene Ontology (GO) study of the DEGs of the microarray resulted in a distribution among the three main functional categories including biological processes (Additional file 5A) with a high presence of genes involved in cell division, cell cycle, cell differentiation and cytoskeleton organization typical of the increased cell proliferating activity during early testicular recrudescence. In addition, genes involved in growth, reproduction, metabolism and catabolism were also differentially expressed. Regarding the molecular function (Additional file 5B), binding, enzymatic activity, and transport were differentially regulated. As for the cell component (Additional file 5C), a majority of the processes appeared taking place in the nucleus and were linked to the protein complex. These results were supported by a GO enrichment analysis that resulted in the identification of several DEGs in the major functional categories undergoing changes throughout European sea bass spermatogenesis (Table 1). Three main subsets were apparent, one including several biological processes focused on reproduction, cell cycle, cell division, chromosome segregation and cellular metabolic processes; a functional subset related to binding; and finally a cellular component category mainly related to processes taking place in the nucleus. The fact that cell cycle processes occur mainly in the nucleus suggests that both subsets are mechanistically related and are involved in cell division and progression. The analysis of the affected biological pathways during the onset of pubertal development (Table 2) indicated that 15 of the DEGs (>2.0 FC) were involved in metabolic pathways mainly related to nucleotide, amino acid and lipid metabolism and retinol metabolism. Pathways involved in cellular processes mainly cell cycle, meiosis, and DNA replication and repair were also affected, and included 13 DEGs. Other group of DEGs was involved in different signalling pathways and the last group includes pathways related with the endocrine system.

**Table 1:** Gene ontology analysis of annotated transcripts significantly affected during the onset of puberty in European sea bass testis (Fisher’s exact test with multiple corrections for FDR)

GO-term	FDR	*P*-value	Sample frequency (*N* = 141)	Array frequency (*N* = 7681)	Gene names
Biological process					
**GO:0007049**. Cell cycle	2.0E-7	2.3E-10	36 (25.5%)	594 (7.7%)	*uhrf2, dmc1, nsl1, ccnd2, kif2c, cenpi,* ***thbs1*** *,* ***jmy*** *, chaf1b, psmd3, ncapg, rbbp4, syce1, ttk, sycp2, pold3,* ***camk2d*** *, ndc80, aspm, rad9b, aurkb, psmb7, bub3, dtymk, ppef1, cenph, anln, slbp, spc25, nup37, mad2l1bp, ccne2, trip13, cdc28, nsmce2, cenpf*
**GO:0051301**. Cell division	3.8E-2	2.2E-3	11 (7.8%)	212 (2.8%)	*nup37, nsmce2, anl, aurkb, cenph, aspm, ndc80, mad2l1bp, bub3, ppef1, cdc28*
**GO:0007059**. Chromosome segregation	2.1E-6	2.1E-08	12 (8.5%)	70 (1.0%)	*nup37, nsmce2, aurkb, cenph, ndc80, kif2c, madl1bp, cenpf, ttk, bub3, syce1, ncapg*
**GO:0006259**. DNA metabolic process	8.0E-3	6.0E-5	20 (14.2%)	401 (5.2%)	*uhrf2, dmc1, cacybp,* ***jmy*** *, chaf1b, pcna, ncapg, rbbp4, pold3, rfc3, rad9b,* ***cry2*** *, smarcc2, asf1b, fen1, trip13, ruvbl2,* ***nt5e*** *, mcm3, nsmce2*
**GO:0010467**. Gene expression	2.0E-7	2.6E-10	4 (2.8%)	1658 (21.6%)	*uhrf2,* ***thbs1*** *, eed, asf1b*
**GO:0000003**. Reproduction	2.3E-2	4.9E-04	14 (9.9%)	266 (3.5%)	***tgfbr1*** *,* ***adamts1*** *, cenpi, bub3, syce1,* ***ihh*** *, sycp2, dmc1, hsf2bp,* ***amh*** *, trip13, sdf1a, ttk,* ***fosla***
**GO:0044237**. Cellular metabolic process	2.1E-3	1.2E-5	47 (33.3%)	3993 (52%)	*uhrf2, dmc1,* ***rab6a*** *, hck, cacybp,* ***thbs1*** *,* ***jmy*** *, chaf1b, pcna, psmd3, ncapg,* ***cry2*** *, gpd1, cad, rbbp4, nme1, ttk, pold3,* ***camk2d*** *, rfc3,* ***lox*** *, srsf7, rad9b, eed,* ***ifitm2*** *, fen1, smarcc2, asf1b, nme3, aurkb, shmt1, cpsf3, atp6v0e1, dtymk, nsmce2, ppef1, u2af35, slbp, trip13,* ***lnx1*** *,* ***fkbp8*** *, nnt, ruvbl2,* ***tgfbr1*** *,* ***nt5e*** *, ubr7, mcm3*
**GO:0050794**. Regulation of cellular process	1.1E-2	8.7E-5	29 (20.6%)	2774 (36.1%)	***bnc1*** *,* ***ihh*** *,* ***agrp2*** *,* ***rab6a*** *,* ***thbs1*** *,* ***c-fosla*** *, psmd3,* ***q2laq1*** *,* ***amh*** *, ect2, cad, trip13,* ***camk2d*** *, ndc80, rad9b,* ***stat3*** *,* ***cry2*** *,* ***sdf1a*** *, ppef1, depdc1b, crabp1, isg20l2, lbr, ap2s1,* ***cyp26a1*** *,* ***fkbp8*** *, tfrc,* ***adamts1*** *,* ***tgfbr1***
Molecular function					
**GO:0043169**. Cation binding	2.4E-3	1.4E-5	8 (5.7%)	1434 (18.7%)	***ihh*** *,* ***mgp*** *,* ***thbs1*** *, cad,* ***tppp3*** *, ppef1,* ***slc25a25*** *,* ***anxa2***
**GO:0043167**. Ion binding	2.4E-3	1.5E-5	8 (5.7%)	1443 (18.8%)	***ihh*** *,* ***mgp*** *,* ***thbs1*** *, cad,* ***tppp3*** *, ppef1,* ***slc25a25*** *,* ***anxa2***
**GO:0046872**. Metal ion binding	3.2E-3	2.1E-5	8 (5.7%)	1414 (18.4%)	***ihh*** *,* ***mgp*** *,* ***thbs1*** *, cad,* ***tppp3*** *, ppef1,* ***slc25a25*** *,* ***anxa2***
Cellular component					
**GO:0005634**. Nucleus	4.8E-2	4.7E-4	55 (39.0%)	2002 (26.1%)	***bnc1*** *, apeh, uhrf2, dmc1,* ***ihh*** *, ccnd2, cacybp, cenpi,* ***jmy*** *,* ***c-fosla*** *, chaf1b, pcna,* ***trhb*** *,* ***sh3bgrl3*** *, rbbp4, syce1, sycp2, pold3,* ***camk2d*** *, rfc3, ndc80, aspm,* ***lox*** *, srsf7, rad9b, eed,* ***stat3*** *,* ***cry2*** *, smarcc2, asf1b,* ***smox*** *, aurkb, shmt1, cpsf3, lbr, psmb7, b9d2, bub3,* ***gfi1*** *, fen1, ass1, ppef1, u2af35, cenph, chrac1, slbp, nup37, cenpf, mad2l1bp, ccne2, trip13, isg20l2, ruvbl2, mcm3, cdc28*
**GO:0005694**. Chromosome	3.9E-7	6.0E-10	25 (17.7%)	302 (3.9%)	*uhrf2, dmc1, nsl1, kif2c, cenpi, pcna, ncapg, rbbp4, syce1, pold3, rfc3, ndc80, rad9b, eed, asf1b, aurkb, bub3, u2af35, spc25, nup37, mad2l1bp, ruvbl2, mcm3, nsmce2, cenpf*
**GO:0000228**. Nuclear chromosome	3.5E-4	1.6E-6	11 (7.8%)	89 (1.1%)	*dmc1, pcna, rbbp4, syce1, pold3, ndc80, rad9b, eed, aurkb, ruvbl2, mcm3*
**GO:0016020**. Membrane	2.0E-7	2.7E-10	14 (9.9%)	2545 (33.1%)	***bnc1*** *,* ***ihh*** *,* ***thbs1*** *,* ***dsg2*** *, cad,* ***camk2d*** *,* ***slco2a1*** *, ppef1, rims1, ap2s1, tfrc,* ***tgfbr1*** *,* ***nt5e*** *,* ***synpo***

Genes in bold type correspond to downregulated genes in the microarray whereas up regulated genes appear in normal type

**Table 2:** Affected KEGG pathways at the onset of European sea bass puberty

Pathway name	Genes involved
Metabolic pathways	
- Nucleotide metabolism	*nme3, pold3, cad, tyms,* ***nt5e*** *, dtymk*
- Retinol metabolism	***cyp26a1***
- Lipid metabolism	***pnpla2*** *, gpd1,* ***agrp2*** *, ptges*
- Amino acid metabolism	*odc1, pycr2,* ***smox*** *, ass1, cad, shmt1,* ***pah***
Cellular processes	
- Cell cycle	*bub3, mcm3, pcna, ttk, ccnd2, mad2l1bp, cdc28, aurkb, ndc80, spc25*
- Meiosis	*mcm3, cdc28, dmc1, mad2l2, sycp2, syce1, cenph, cenpi, cenpf, ndc80, spc25*
- DNA replication and repair	*mcm3, pold3, fen1, pcna, rfc3*
- Focal adhesion	***actb*** *, ccnd2,* ***thbs1***
Genetic information processing	
- FoxO signalling pathway	***tgfbr1*** *,* ***stat3*** *, plk4, ccnd2*
- Hippo signalling pathway	***amh*** *,* ***tgfbr1*** *, ccnd2,* ***actb***
- Jak-STAT signalling pathway	***stat3*** *,* ***socs3*** *, ccnd2*
- TGF beta signalling pathway	***amh*** *,* ***tgfbr1*** *,* ***thbs1***
- MAPK signalling pathway	*gpd1, cdc28,* ***tgfbr1***
- TNF signalling pathway	***socs3***
- Toll-like receptor signalling pathway	*ctsk*
- Wnt signalling pathway	*cacybp, ccnd2*
- PI3K-Akt signalling pathway	*ccnd2,* ***thbs1***
- cAMP signalling pathway	*adcyap1,* ***amh***
- Hedgehog signalling pathway	*ccnd2, ihh*
- Rap1 signalling pathway	*actb, thbs1*
Endocrine system	
- Prolactin signalling pathway	***stat3*** *,* ***socs3*** *, ccnd2*
- Renin secretion	***aqp1***
- Thyroid hormone signalling pathway	***actb***

Downregulated genes appear in bold type whereas upregulated genes appear in normal type

### Microarray validations

**Fig. 4:**
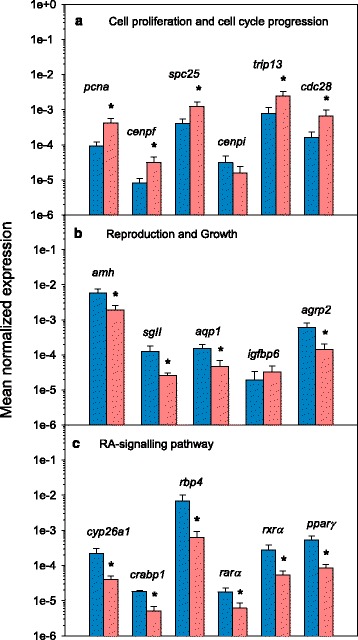
qPCR results for genes differentially expressed in the microarray during the onset of puberty in the European sea bass. Genes were selected according to their relevance in different reproductive events. **a** Genes involved in cell proliferation and cell cycle progression; proliferating cell nuclear antigen (*pcna*), centromere protein I (*cenpi*), spindle pole body component 25 (*spc25*), centromere protein f (*cenpf*), thyroid hormone receptor interactor 13 (*trip13*), and cdc28 protein kinase (*cdc28*). **b** Genes involved in reproduction and growth; antimüllerian hormone (*amh*), aquaporin 1 (*aqp1*), secretogranin II (*sgII*), agouti-related protein 2 (*agrp2*), insulin-like growth factor binding protein 6 (*igfbp6*). **c** Genes involved in the RA signalling pathway: RA-metabolizing enzyme cytochrome P450 26a1 (*cyp26a1*), retinol binding protein 4 (*rbp4*), RA-binding protein (*crabp1*). This group also includes three RA-nuclear receptors, RA receptor alpha (*rarα*), retinoid X receptor alpha (*rxrα*), peroxisome proliferator-activated receptor gamma (*pparγ*) due to their relevance in this pathway. The stage-specific expression levels were normalized to those of the constitutively expressed *18S rRNA* gene in each sample. Expression data are shown as mean normalized expression + SEM. Y-axis is represented in logarithmic scale for easier visualization. For each gene, bars on the left (*blue*) correspond to stage I testes and bars on the right (*red*) to stage II testes

**Fig. 5:**
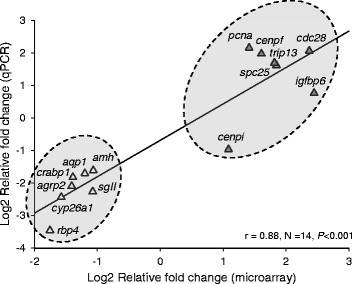
Correlation between microarray and qPCR results. Fold change (FC) induction values of the different gene transcripts are plotted as log2 values of the relative fold change. The X-axis represent microarray data whereas the Y axis corresponds to qPCR data. The regression line and the corresponding r coefficient are also represented. For gene symbols and complete gene names see caption of Fig. 4

For qPCR validations of the microarray results, several DEGs representing different categories of interest were selected according to their relevance in reproduction. All of them were cloned and sequenced to confirm their identity. The relative differential expression was assessed for 14 transcripts. Six of them (*pcna*, *cenpi*, *spc25*, *cenpf*, *trip13*, *cdc28*), were included in a group of genes with special relevance in cell proliferation and cell cycle progression (Fig. 4a). Five transcripts (*aqp1*, *amh*, *sgII*, *agrp2, igfbp6*) were included in the group of genes with relevance in reproduction and growth (Fig. 4b). The remaining three transcripts (*cyp26a1*, *rbp4*, *crabp1*) were located in the group of genes involved in the RA-signalling pathway (Fig. 4c). In addition, and due to their prominent role in that pathway, the expression of three more transcripts, corresponding to RA-nuclear receptors (*rarα*, *rxrα* and *pparγ*) was studied in stage I and stage II testis (Fig. 4c). The stage-specific expression levels were normalized to those of the constitutively expressed *18S rRNA* gene in each sample. The qPCR results were consistent and showed a good correlation with those of the microarray data (Fig. 5). It is worth mentioning that in a number of genes, the microarray results exhibited lower differences between the two developmental stages than those found from the qPCR, indicating that this particular microarray may represent an underestimate of the extent of differential expression during European sea bass spermatogenesis (Additional file 6).

### Molecular cloning of European sea bass *cyp26a1* and phylogenetic analysis

**Fig. 6:**
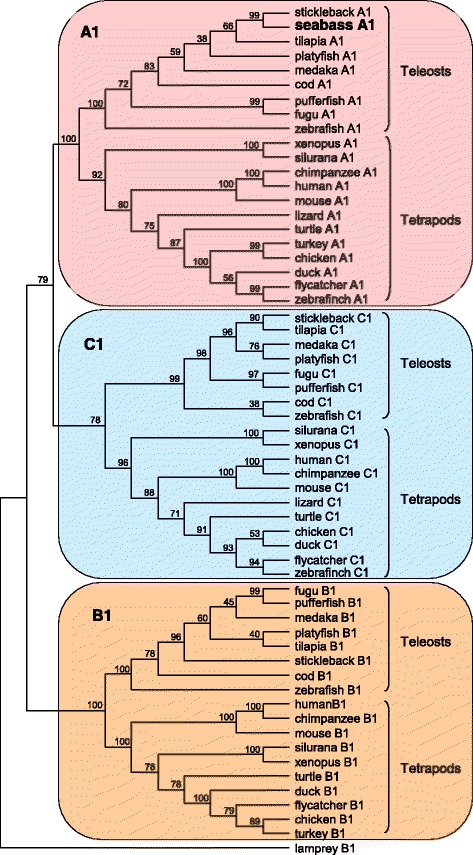
Phylogenetic tree of Cyp26 family proteins. The tree was constructed using the Neighbor-Joining method. The bootstrap consensus tree inferred from 1000 replicates is taken to represent the evolutionary history of the taxa analysed. The percentage of times each branching was obtained out of the 1000 bootstraps is shown next to the nodes. The evolutionary distances were computed using the Poisson correction method. All positions containing alignment gaps and missing data were eliminated in pairwise sequence comparisons (pairwise deletion option). Phylogenetic analyses were conducted in MEGA4 [49]. A cyp26b1-like isoform from lamprey was used as outgroup to root the tree. GenBank/Ensembl accession numbers of the sequences used to generate the tree appear listed in Additional file 7

Among the DEGs found in the microarray we focused on *cyp26a1* due to its prominent role in the RA signalling pathway by maintaining the homeostasis of intracellular RA levels [50] and because RA is known to be essential for the onset of meiosis in several vertebrates [51–54]. The cDNA isolated for European sea bass *cyp26a1* contained an ORF 1178 bp long, was flanked by a 500 bp 3’UTR region and was deposited in the GenBank under the accession number KJ187657. The deduced amino acid sequence encodes a protein 488 amino acid long with a theoretical PI of 9.04 and a calculated molecular weight of 55.495 kDa. A Genbank search resulted in the identification of several full-length sequences for Cyp26 proteins in teleosts and tetrapods, including amphibians, reptiles, birds and mammals. The phylogenetic analysis showed that the European sea bass protein was evolutionarily closer to CYP26A1 proteins, while it was more distant from other CYP26B1 and CYP26C1 proteins (Fig. 6 and Additional file 7 for accession numbers). The consensus tree had three main branches, one containing all A1 sequences, another one including B1 sequences and the remaining with C1 sequences. Sea bass sequence clustered together in the A1 group further supporting its identity. Comparisons of the deduced amino acid sequence with other full-length Cyp26a1 in different fish revealed that the highest homology was shared with stickleback (94.4%) and the lowest with zebrafish (82.4%). These slight differences among fish support that Cyp26a1 in teleosts is quite highly conserved. A study of the 5′ flanking sequence (1500 bp upstream of the first ATG) of the *cyp26a1* gene showed the presence of binding sites for different transcription factors among which it is worth mentioning RA-nuclear receptors (Rxr, Rar, and Ppar), steroid receptors, and several elements involved in cell cycle regulation (Additional file 8).

### Tissue specific expression

**Fig. 7:**
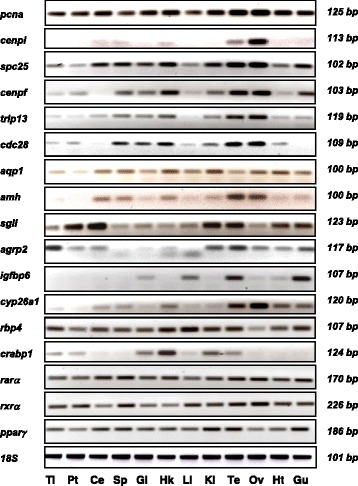
Tissue distribution of European sea bass transcripts involved in different biological processes during sea bass early puberty. Biological processes include: cell proliferation and cell cycle progression (*pcna*, *cenpi*, *spc25*, *cenpf*, *trip13*, and *cdc28*); reproduction (*aqp1*, *amh*, *sgII*, *agrp2*, and *igfbp6*; RA signalling pathway (*cyp26a1*, *rbp4*, *crabp1*, *rarα*, *rxrα*, and *pparγ*). The expression was detected by RT-PCR in different tissues including telencephalon (Tl), pituitary (Pt), cerebellum (Ce), spleen (Sp), gills (Gi), head kidney (Hk), liver (Li), posterior kidney (Ki), testis (Te), ovary (Ov), heart (Ht), and gut (Gu). 18S ribosomal RNA (*r18S*) was used as a positive internal control to check for the integrity of the cDNA template. For complete gene names see caption of Fig. 4

The expression of the selected DEGs and the three extra genes in different tissues was assessed by conventional PCR (Fig. 7). Among the cell proliferation genes, *pcna* and *spc25* were ubiquitously expressed whereas *cenpi* expression was mainly restricted to gonads, with highest levels in ovary. Regarding the genes involved in reproduction and growth, *aqp1* was expressed at similar levels in all the tissues studied. *sgII* expression was found in all tissues but with higher levels in pituitary, cerebellum, kidney and testis. The expression of *amh* was highest in gonads and undetectable in telencephalon and pituitary whereas *igfbp6* expression was restricted to the gills, liver, testis and gut. Finally, the genes involved in RA-signalling pathway were expressed at similar levels in all tissues except *cyp26a1* that showed highest expression in gonads and *crabp1* that was mostly expressed in head kidney and at lower levels in dorsal kidney, gills and testis.

## Discussion

The present study contributed to identify differences in gene expression during the early stages of pubertal development in European sea bass males using a custom-made microarray. However, and despite the fact that the differences observed at the transcriptome and the steroid level are related to puberty (first successful reproduction), it is possible that similar changes could be found in successive reproductive seasons since they mark the transition between quiescence and the start of gametogenesis. The use of long oligo-based microarrays has been shown to have a higher sensitivity for detection but a lower specificity [55] and therefore could have a reduced ability to discriminate between similar transcripts produced by the same locus, paralogs or similar members of large gene families. To circumvent this problem, we cloned the full-length sequence of several selected DEGs and then validated the results with qPCR obtaining a good correlation between both methods. In addition, the study revealed the complete sequences of some transcripts for the first time in the European sea bass, adding contrasted information to the microarray that was based on EST sequences. However, for other DEGs that were not annotated in the microarray, we could not find any match to reveal their identities in any of the databases searched including Genebank, Ensembl, and Uniprot. The different stages of spermatogenesis (stage I and stage II) used for the study were classified by histology and their corresponding 11KT levels were further confirmed by EIA, demonstrating that the increase of circulating 11KT marks the initiation of pubertal development. A similar result was found in other teleosts including eel [56], goldfish [57], zebrafish [16] or trout [58] and 11KT measured from the mucus of carps was suitable to differentiate males from females [59, 60]. However, no correlation between 11KT and gonad developmental stage in either male or female carp could be found [59]. Our results open the possibility to explore the use of 11KT as a non-lethal marker for the onset of puberty in this species helping to manage the fish farms stocks to separate precocious from non-precocious European sea bass males.

The transcriptome response revealed that cell proliferation, cell cycle and meiosis progression were pathways preferentially affected during the onset of male puberty. The spindle assembly checkpoint (SAC) is a control mechanism of dividing cells that ensures the correct segregation of chromosomes by blocking cell cycle progression until kinetochores are properly connected to the spindle [61]. In our study, several genes coding for SAC proteins (*bub3*) and SAC protein regulators (*mad2l1bp* and *ttk*), were upregulated in stage II testes as well as other important kinetochore-associated transcripts such as *ndc80*, *spc25*, *aurkb* and *cdc28* (Additional file 2). *ndc80* and *spc25* code for essential proteins of the Ndc80 complex, needed for SAC activity [62] while *aurkb* (aurora kinase b) controls kinetochore orientation during meiosis [63]. Defects in *cdc28* function result in delays in the exit from mitosis and in meiosis impairment among others [64]. Transcripts coding for centromere proteins like *cenph*, *cenpi* or *cenpf*, were also upregulated. In the case of *cenpi*, in addition to its role in centromere formation, it is involved in the response of gonadal tissues to Fsh [65]. The upregulation of *cenpi* in stage II coincides with the initiation of the gradual increase in Fsh plasma levels in European sea bass during early spermatogenesis [1, 33]. This is in line with the role of Fsh inducing germ cell proliferation and marks the onset of spermatogenesis through the activation of spermatogenesis-related genes [34]. Several transcripts like *sycp2* and *syce1*, coding for proteins of the synaptonemal complex and *trip13*, required for the completion of meiosis [66], were upregulated in stage II testes. Moreover, *pcna*, essential for DNA replication and a molecular marker of dividing cells [67], also increased during the onset of spermatogenesis in agreement with the active mitosis of spermatogonia typical of this period [18]. The role of *pcna* in the proliferation of germ cells has been described in several teleosts and is currently used in a number of fish species as a marker of spermatogenesis progression [2]. All these results are supported by the analysis of affected biological pathways (Table 2) that showed higher expression of genes involved in cellular processes, particularly those involved in cell cycle, meiosis and DNA replication and repair. In addition, several signalling pathways involved in testicular development such as Wnt, MAPK, hedgehog and TGF beta signalling pathways [68] were altered during early puberty in European sea bass. Altogether, the upregulation of the above mentioned genes is indicative of an active period of mitosis, reflect the need for a tight control of the correct division of the cells, and constitutes an indicator for the progression of meiosis typical of this stage.

A second group of DEGs includes those implicated in reproduction and growth. *Amh* is involved in gonadal development and steroidogenesis in vertebrates and induces the regression of müllerian ducts in mammals during male embryogenesis [69]. Although fish do not have müllerian ducts, *amh* homologues have been identified in several teleosts [70], suggesting evolutionary conserved functions for this gene. The role of Amh as a meiosis inhibiting factor was first shown in eels [71] and recently in zebrafish [16], induced by the increase of circulating 11KT that blocked *amh* expression facilitating spermatogenesis completion. In teleosts, *amh* has a key role in early testicular maturation with highest levels in pre-spermatogenic testis and lowest during spawning [72]. In European sea bass, administration of recombinant Fsh induced spermatogonial proliferation and differentiation into spermatocytes, due to the increase of 11KT levels and the concomitant suppression of *amh* expression [34]. Moreover, *amh* mRNA and protein expression was detected in Sertoli cells of prepubertal European sea bass, and the signal decreased during spermatogenesis [73]. Our results showed a decrease of *amh* levels during early spermatogenesis in agreement with its role as an inhibitor of spermatogenesis progression. This is supported by the increase of *pcna*, *cenpi* and 11KT levels in the same testicular stage. Likewise, a downregulation of *amh* during the reproductive cycle has been found in the testicular transcriptome of rainbow trout [12] and in precocious Atlantic salmon [74], further demonstrating that the inhibitory effect of Amh of the onset of puberty can be extended to all fish species so far studied. In addition, the study of the biological pathways affected during the onset of puberty, also show the importance of *amh* in several signalling pathways including those of TGF beta, hippo and cAMP [68]. Few studies are available for *sgII* in fish, apart from those in goldfish [75–77]. SgII is widely distributed in secretory granules of neurons and endocrine cells [78] and is the precursor of secretoneurin, a bioactive neuropeptide capable to induce Lh secretion [75, 77]. Our results show low *sgII* expression by the onset of spermatogenesis, in agreement with the low Lh levels found at this stage in the European sea bass [1, 30]. It would be very interesting to determine whether *sgII,* and therefore secretoneurin, also increase during later stages of spermatogenesis, coinciding with the surge of Lh, to experimentally test this hypothesis. Pioneering studies in sea bream point at the relevance of aquaporin 1 (aqp1) in fish reproduction due to its role in water intake during oocyte hydration prior to spawning [79] and in the activation of sperm motility during the last stages of spermatogenesis [80, 81]. Our results show low *aqp1* levels during early spermatogenesis, in agreement with its prominent role in sperm maturation during the last stages of spermatogenesis. Agrp2 (agouti-related protein 2) is an orexigenic peptide with a key role in the regulation of energy balance in mammals [82] and fish [83]. In this regard, our study shows that the lipid metabolism pathway where *agrp2* was included was affected during the early stages of pubertal development further supporting its role in energy balance. A direct link between leptin, the most powerful orexigenic neuropeptide in fish [84], and the AGRP system has been suggested in European sea bass males [8]. Moreover, abundant *Agrp* expression was found in mouse pachytene-spermatocytes and immunohistochemistry revealed that Agrp co-localized with Scp3, a meiotic-specific protein of the synaptonemal complex [85]. Although *agrp2* has been characterized in European sea bass testis [86], this is the first time its involvement in spermatogenesis is suggested, possibly due to the specific energy requirements during spermatogenesis and the decrease in food intake. This is in agreement with the downregulation of *agrp2* in European sea bass brain after long-term fasting [86] and its decrease in testis during early spermatogenesis (present study), and link the appetite and growth system with reproduction [87]. Moreover, a transcriptomic study of trout testis revealed that Fsh administration induced the increase of *igfbp6* [88]. This strong Fsh-induced upregulation was present during early spermatogenesis, including germ cell proliferation and meiosis, and was associated to the effect of the Igf-signalling pathway on spermatogenesis progression [89]. Our results also show a clear upregulation of *igfbp6*, coinciding with the first stages of pubertal development and the increase of 11KT plasma levels.

The last group of DEGs is associated with the RA-signalling pathway. RA has been proposed as a meiosis inducing factor in tetrapods including mammals [51], birds [52], amphibians [53] and fish [4, 5, 54, 90]. Two transcripts coding for binding proteins, one in charge of retinol transport (*rbp4*) through the blood stream and another one (*crabp1*) in charge of the translocation of RA to the nucleus of the target cells [91] were differentially expressed in European sea bass transcriptome. In addition, *cyp26a1*, responsible for the degradation of intracellular RA and essential for the maintenance of RA homeostasis [50], was affected. The decrease of *cyp26a1* in stage II is associated with a decrease in the translocation and transport of RA brought about by the downregulation of *rbp4* and *crabp1*, in order to maintain the homeostasis of RA that otherwise, and at high levels can be toxic for the cell [92]. In addition, retinol metabolism was one of the affected metabolic pathways found in the present study. It seems thus plausible that in European sea bass, the suppression of RA degradation and the concomitant increase in the availability of RA could be partially responsible for triggering the onset of meiosis. In zebrafish testes, *cyp26a1* was expressed in germ cells entering meiosis, while in females, a downregulation was found in oocytes during meiosis resumption [54]. Likewise, in medaka, RA was found to act directly on Sertoli cells, Leydig cells, and pre-meiotic germ cells with a decrease of *cyp26a1* expression by the time of meiosis resumption, whereas in ovaries, RA-transcriptional activity is highest in meiotic oocytes [5]. In addition, in vivo Fsh-injection to pre-spermatogenic zebrafish males induced the onset of spermatogenesis and resulted in changes of several enzymes involved in the RA-signalling pathway, including a decrease in *cyp26a1* expression, although no effect was found after ex vivo culture of pre-spermatogenic testes with Fsh [17]. Moreover, the administration of an inhibitor of RA synthesis in combination with a deficient diet of vitamin A (a precursor of RA) to adult zebrafish also induced a downregulation of *cyp26a1*, most likely to increase intracellular RA levels, although spermatogenesis was still disrupted, and fertility compromised [93]. The above mentioned studies suggest that a decrease in *cyp26* expression is associated with the onset of spermatogenesis and the initiation of meiosis and prompted us to clone and obtain the full length of its cDNA. The alignment of the deduced protein sequence with other Cyp26 proteins available from other vertebrates confirmed its identity as Cyp26a1 revealing slight differences in homology among teleosts, further supporting their high conservation due to its pivotal role controlling RA levels. The tree shows the presence of a common ancestor cyp26 protein, strengthening the hypothesis of an independent functionalization of its coding gene prior to the two rounds of genome duplication in vertebrates [54]. Moreover, the study of the promoter showed the presence of binding sites for cell cycle regulators and for RA nuclear receptors including Ppar, Rxr and Rar, and also DR1 and DR5 sites (RA-responsive elements) indicating the role of RA, via the interaction with its nuclear receptors, in the regulation of *cyp26a1* transcription in the European sea bass. A similar result has been shown for the *cyp26a1* promoter in zebrafish [94, 95] and medaka [5], although the development of functional studies is clearly needed to confirm the capability of RA to induce the regulation of *cyp26a1* in the European sea bass. To gain more insight on the importance of the RA-signalling pathway in meiosis we studied the expression of several nuclear receptors involved in RA binding including *rarα*, *raxrα*, and *pparγ*. The receptors appeared ubiquitously expressed and at similar levels in all the tissues studied, reflecting the general actions and the importance of RA in numerous biological processes throughout evolution [96] and its involvement in the proliferation and differentiation of many cell types [97].

## Conclusion

To the best of our knowledge, this is the first transcriptomic study focussed on the early stages of puberty, and aimed at the identification of molecular and endocrine signals triggering the start of the initial spermatogenic wave in European sea bass. Increases in androgen plasma levels, particularly 11KT, mark the transition between testicular stage I and stage II. This opens the possibility to explore the use of 11KT in the management of European sea bass stocks in aquaculture farms to separate precocious from non-precocious males. The study improved the annotation of different genes of the microarray and helped to increase the knowledge of several mechanisms and biological pathways involved in early stages of puberty. Altogether, the study shows that the onset of spermatogenesis is characterized by the activation of genes involved in cell cycle progression and division including mitosis and meiosis. The differential expression of several components of the RA-signalling pathway suggests their important role in the onset of meiosis. This work lays the foundation for an in-depth study of the RA-signalling signalling pathway and its role in the onset of meiosis in fish. A future increase in the sequencing of the European sea bass gonad transcriptome and the use of RNA-seq technologies will help to shed light on the molecular pathways involved in relevant aspects of the reproductive process of this economically important species and will aid to develop comparative studies on gonadal differentiation and maturation in teleosts.

## Additional files

Additional file 1: A table containing qPCR primer characteristics and different features to calculate the efficiency of the primers in the amplification reactions (word format, .doc). (DOC 52 kb)

Additional file 2: A table containing all the DEGs found in the microarray during the onset of European sea bass puberty. The searchable excel file contains the probe ID and primary accession numbers assigned in the custom European sea bass microarray to all differentially expressed genes. The *p*-value and adjusted *p*-value are also included, as well as the fold change and the regulation (stage I versus stage II) obtained after comparison of sea bass testes during the onset of puberty. The annotation information is given in the gene description column that reported the presence of 153 non-annotated sequences from a total of 315 differentially expressed genes, and in the gene description column containing a brief explanation of each gene function. Finally, a column with the assigned GO-terms is also included (excel format, .xls). (XLS 144 kb)

Additional file 3: Gene abbreviation glossary of the most relevant DEGs found in the microarray during the onset of European sea bass puberty. A table containing all the DEGs that appear specifically mentioned in the study out of the 315 DEGs found after microarray hybridizations and qPCR validations (word format, .doc). (DOC 51 kb)

Additional file 4: Principal component analysis of the transcriptomic results from the microarray hybridizations. Each data point corresponds to a pool of RNAs from the testis of six different fish. Blue circles correspond to RNAs from testis in developmental stage I and red circles to RNAs from testis in stage II. Numbers between brackets represent the percentage of variation explained by each component; i.e. component 1 (PC1) and component 2 (PC2) (powerpoint format, .ppt). (PPT 108 kb)

Additional file 5: Distribution by GO-terms of the differentially expressed genes (DEGs) during the onset of European sea bass puberty. The multi-level pie graph classified the DEGs according to their GO-terms in three main categories, including: A; Biological processes (cutoff value = 10 sequences), B; molecular function (cutoff value = 5 sequences), and C; cell component (cutoff value = 5 sequences). The number of genes found in each GO-term appear written between parentheses (powerpoint format, .ppt). (PPT 147 kb)

Additional file 6: A table containing the microarray versus qPCR fold change (FC) expression values for 14 genes differentially expressed in the European sea bass transcriptome during early stages of pubertal development (word format, .doc). (DOC 54 kb)

Additional file 7: A table containing all the protein sequences used to generate the phylogenetic tree for cyp26 (word format, .doc). (DOCX 16 kb)

Additional file 8: Transcription factor binding sites along the European sea bass *cyp26a1* promoter sequence (1500 bp). The searchable excel file contains different columns reporting information of the family of transcription factor, information on the matrix, the position of the binding site in the sequence (start and end of the sequence), the identification of the DNA strand (+ or -), the similarity of the core and the matrix sequences and finally the sequence (excel format, .xls) (XLS 39 kb)
