# Erratum to: Identification of conserved genes triggering puberty in European sea bass males (*Dicentrarchus labrax*) by microarray expression profiling

**DOI:** 10.1186/s12864-017-3909-x

**Published:** 2017-07-05

**Authors:** Mercedes Blázquez, Paula Medina, Berta Crespo, Ana Gómez, Silvia Zanuy

**Affiliations:** 1 Instituto de Acuicultura de Torre la Sal, Consejo Superior de Investigaciones Científicas (IATS-CSIC), Ribera de Cabanes, 12595 Castellón, Spain; 2 Instituto de Ciencias del Mar, Consejo Superior de Investigaciones Científicas (ICM-CSIC), Passeig Maritim 37-49, 08003 Barcelona, Spain; 3 Present address: Universidad de Antofagasta, Avda Angamos, 601 Antofagasta, Chile; 4 Present address: UCL Great Ormond Street Institute of Child Health, 30 Guilford Street, London, WC1N 1EH UK

## Erratum

After publication of the original article [1] the authors found that additional funding had not been disclosed for Professor Silvia Zanuy from Generalitat Valenciana (Prometeo II/2014/051) in the ‘**Funding**’ section.

The correct Funding information should therefore be the below (additional information is included in bold):

**Funding**

Work supported by Aquagenomics project (CSD2007–0002) from **the Spanish Government to SZ, by project (PrometeoII/2014/051) from the Generalitat Valenciana to SZ** and by a MICINN project (AGL2011–28890) to AG. PM was supported by the University of Antofagasta grant MECE2 (ANT0806).
