# Supplementary material for: Identification of conserved genes triggering puberty in European sea bass males (Dicentrarchus labrax) by microarray expression profiling
Source: BMC Genomics. 2017 Jun 5;18:441. doi: 10.1186/s12864-017-3823-2 (PMC5460432; doi:10.1186/s12864-017-3823-2)
Supplement: Supplementary file 1 — A table containing qPCR primer characteristics and different features to calculate the efficiency of the primers in the amplification reactions (word format, .doc). (DOC 52 kb) [file 12864_2017_3823_MOESM1_ESM.doc]

Additional file 1. Quantitative real time PCR (qPCR) primers and features to calculate the efficiency

| **Gene symbol** | **Accesion number** | **Amplicon length (bp)** | **Primer sequences (5’→3’)** | **Efficiency (%)** | **Slope** | **R^2^** |
| --- | --- | --- | --- | --- | --- | --- |
| ***pcna*** | JQ755266 | 125 | Fwd: CCAAGGACGGAGTCAAGTTC  Rev: CTGGACGGGTTCATTCATCT | 99 | -3.37 | 0.99 |
| ***cenpi*** | KP739864 | 113 | Fwd: TCTGGCTAGGCTACGCTCTC  Rev: GTGCTGTGTGGACAGCAACT | 101.8 | -3.24 | 0.99 |
| ***spc25*** | KP729615 | 102 | Fwd: CATTTGGGGATGGAGATACG  Rev: GTCCTGATCCAAAGGGTTGA | 96.5 | -3.50 | 0.98 |
| ***cenpf*** | KP765683 | 103 | Fwd: GGCAGCTTGACAAGATCACA  Rev: GAGAGACGGCACACTTCTCC | 97.3 | -3.45 | 0.96 |
| ***trip13*** | KP739862 | 119 | Fwd: GGGATTTGGGAGAGTCTGGT  Rev: CGGTTCCATGAAATCAGGTT | 93.5 | -3.68 | 0.99 |
| ***cdc28*** | KP729616 | 109 | Fwd: GAGGAAGAGTGGAGGGGACT  Rev: CCTTTGGGAGAGGTCTTCTG | 96.3 | -3.51 | 0.98 |
| ***igfbp6*** | KP739865 | 107 | Fwd: CCAACTGAAAACGTCCAAACG  Rev: GGCGTCAAGACCTTTGATAAGTGTA | 99.9 | -3.33 | 0.98 |
| ***cyp26a1*** | KJ187657 | 120 | Fwd: GCAGGAGCTGGTGGAAGCTT  Rev: CCTTGCCTTCAGACCCCTGTA | 100.6 | -3.29 | 0.99 |
| ***rbp4*** | KP739863 | 107 | Fwd: ACCCTGCCAAGTTCAGAATG  Rev: GTGGACGGCGTAGTTATCGT | 97.6 | -3.44 | 0.99 |
| ***crabp1*** | KP723829 | 124 | Fwd: GCCACTTGGGAAACAGAAAA  Rev: CATCGGCTCCAAAGATCAGT | 99.2 | -3.36 | 0.96 |
| ***aqp1*** | DQ924529 | 100 | Fwd: TTGGCAGCAATCAGCTACAC  Rev: CCCAGTACACCCAGTGGTCT | 99.1 | -3.36 | 0.99 |
| ***amh*** | AM232701 | 100 | Fwd: TGACTCCACTTCTGCTTTTCTCAT  Rev: AGAAAGGAGGAGGTCTGTGAAGAG | 93.5 | -3.68 | 0.93 |
| ***sgII*** | KP729617 | 123 | Fwd: ACGAGGTGACCACCTACCTG  Rev: CGTACAGCTCGTAGGGGAAC | 101.7 | -3.24 | 0.95 |
| ***agrp2*** | HE660087 | 117 | Fwd: GGGCAGAGGACACAAAGAAA  Rev: TGTGACTTTCCTGTGGTGGA | 98.1 | -3.42 | 0.99 |
| ***rarα*** | KP749835 | 170 | Fwd: CGCTAAACCGAACCCAGA  Rev: CTTCTCGGCCTGTTCCAA | 100.9 | -3.28 | 0.98 |
| ***rxrα*** | KP749834 | 226 | Fwd: CTGGTAGAGTGGGCCAAGAG  Rev: GTTCTGTGAGCACCCTGTCA | 97.8 | -3.43 | 0.98 |
| ***pparγ*** | AY590303 | 186 | Fwd: CAGATCTGAGGGCTCTGTCC  Rev: CCTGGGTGGGTATCTGCTTA | 97.2 | -3.46 | 0.99 |
| ***r18S*** | AY831388 | 101 | Fwd: CCGCTTTGGTGACTCTAGATAACC  Rev: CAGAAAGTACCATCGAAAGTTGATAGG | 100 | -3.30 | 0.99 |
