# Supplementary material for: Identification of conserved genes triggering puberty in European sea bass males (Dicentrarchus labrax) by microarray expression profiling
Source: BMC Genomics. 2017 Jun 5;18:441. doi: 10.1186/s12864-017-3823-2 (PMC5460432; doi:10.1186/s12864-017-3823-2)
Supplement: Supplementary file 3 — Gene abbreviation glossary of the most relevant DEGs found in the microarray during the onset of European sea bass puberty. A table containing all the DEGs that appear specifically mentioned in the study out of the 315 DEGs found after microarray hybridizations and qPCR validations (word format, .doc). (DOC 51 kb) [file 12864_2017_3823_MOESM3_ESM.doc]

Additional file 3. Glossary of genes involved in cell proliferation, reproduction, growth and RA-signalling pathway with particular mention in the study

| Gene symbol | Gene name | |
| --- | --- | --- |
| *agrp2* | Agouti-related protein 2 |  |
| *amh* | Anti-Müllerian hormone |  |
| *aqp1* | Aquaporin 1 |  |
| *aurkb* | Aurora kinase b |  |
| *bub3* | Mitotic checkpoint protein bub3 |  |
| *cdc28* | Cell division control protein 28 |  |
| *cenpf* | Centromere protein F |  |
| *cenph* | Centromere protein H |  |
| *cenpi* | Centromere protein I |  |
| *crabp1* | Cellular retinoic acid binding protein 1 |  |
| *cycl1* | Cylicin 1 |  |
| *cyp26a1* | Cytochrome P450, family 26, subfamily A, member 1 |  |
| *igfbp6* | Insulin-like growth factor binding protein 6 |  |
| *mad2l1bp* | Mitotic arrest deficient-like 1 (mad2l1) binding protein |  |
| *ndc80* | Ndc80 kinetochore complex component |  |
| *pcna* | Proliferating cell nuclear antigen |  |
| *pparγ* | Peroxisome proliferator activated receptor γ |  |
| *rarα* | Retinoic acid receptor alpha |  |
| *rbp4* | Retinol binding protein 4 |  |
| *rxrα* | Retinoid X receptor gamma |  |
| *sgII* | Secretogranin II |  |
| *spc25* | Spc25, kinetochore complex component |  |
| *sycp2* | Synaptonemal complex protein 2 |  |
| *trip13* | Thyroid hormone receptor interactor 13 |  |
| *ttk* | ttk protein kinase |  |
