# Supplementary figures and images for: Identification of conserved genes triggering puberty in European sea bass males (Dicentrarchus labrax) by microarray expression profiling
Source: BMC Genomics. 2017 Jun 5;18:441. doi: 10.1186/s12864-017-3823-2 (PMC5460432; doi:10.1186/s12864-017-3823-2)

## Slide 1
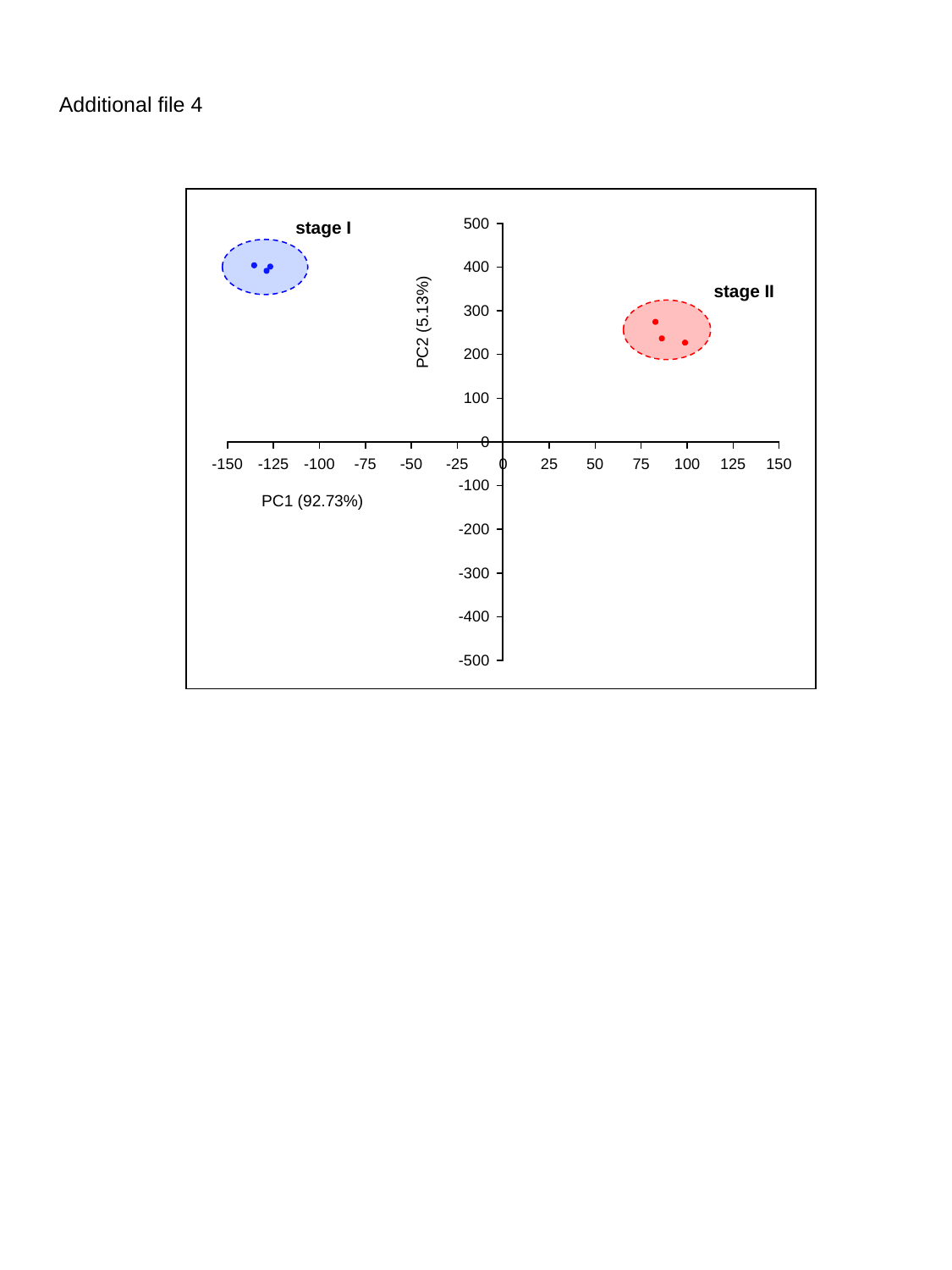

Additional file 4
stage I
stage II

Supplement: Supplementary file 4 — Principal component analysis of the transcriptomic results from the microarray hybridizations. Each data point corresponds to a pool of RNAs from the testis of six different fish. Blue circles correspond to RNAs from testis in developmental stage I and red circles to RNAs from testis in stage II. Numbers between brackets represent the percentage of variation explained by each component; i.e. component 1 (PC1) and component 2 (PC2) (powerpoint format, .ppt). (PPT 108 kb) [file 12864_2017_3823_MOESM4_ESM.ppt]
