# Supplementary material for: Identification of conserved genes triggering puberty in European sea bass males (Dicentrarchus labrax) by microarray expression profiling
Source: BMC Genomics. 2017 Jun 5;18:441. doi: 10.1186/s12864-017-3823-2 (PMC5460432; doi:10.1186/s12864-017-3823-2)
Supplement: Supplementary file 5 — Distribution by GO-terms of the differentially expressed genes (DEGs) during the onset of European sea bass puberty. The multi-level pie graph classified the DEGs according to their GO-terms in three main categories, including: A; Biological processes (cutoff value = 10 sequences), B; molecular function (cutoff value = 5 sequences), and C; cell component (cutoff value = 5 sequences). The number of genes found in each GO-term appear written between parentheses (powerpoint format, .ppt). (PPT 147 kb) [file 12864_2017_3823_MOESM5_ESM.ppt]

## Slide 1
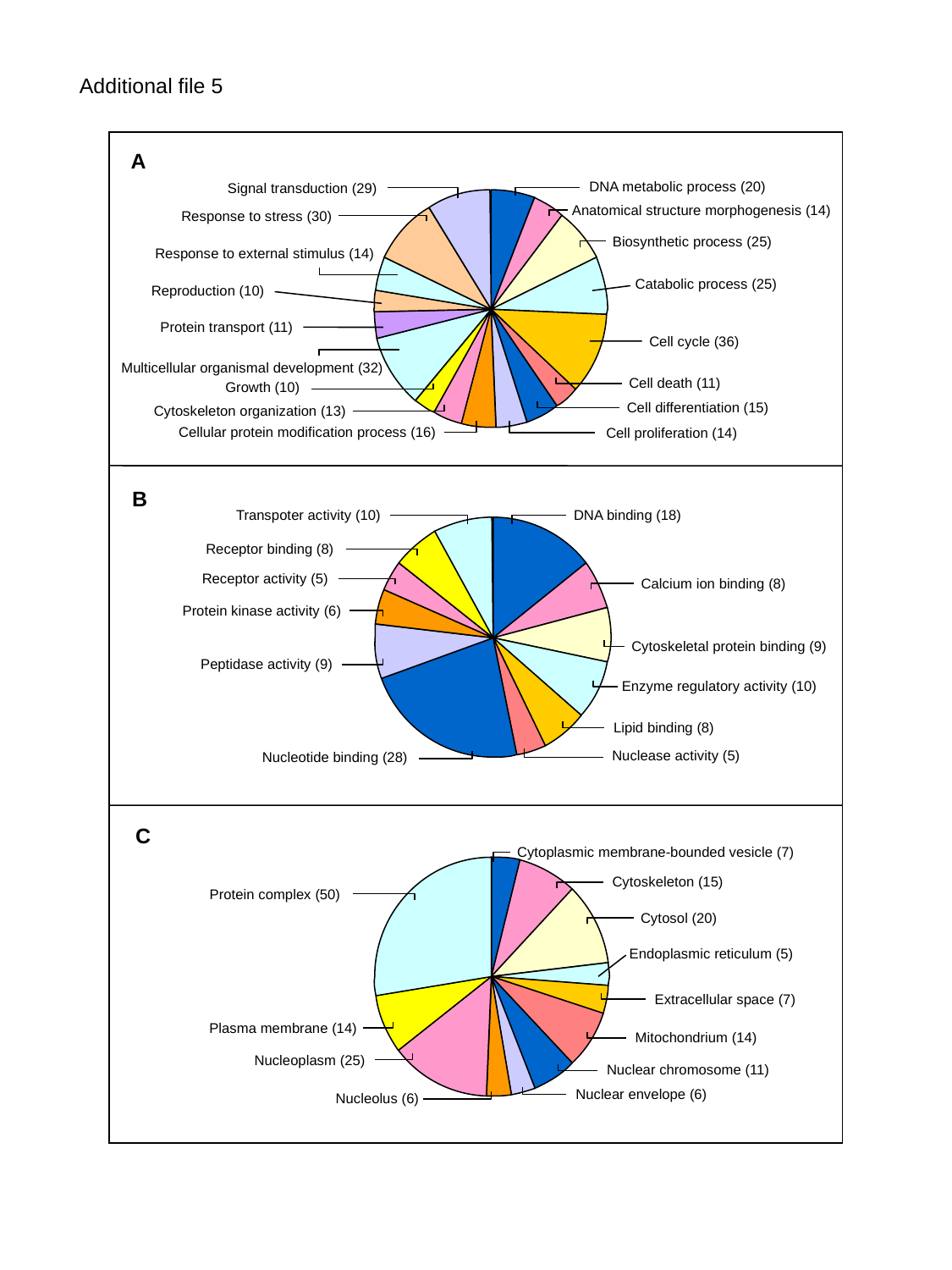

Additional file 5
A
DNA metabolic process (20)
Signal transduction (29)
Anatomical structure morphogenesis (14)
Response to stress (30)
Biosynthetic process (25)
Response to external stimulus (14)
Catabolic process (25)
Reproduction (10)
Protein transport (11)
Cell cycle (36)
Multicellular organismal development (32)
Cell death (11)
Growth (10)
Cell differentiation (15)
Cytoskeleton organization (13)
Cellular protein modification process (16)
Cell proliferation (14)
B
DNA binding (18)
Transpoter activity (10)
Receptor binding (8)
Receptor activity (5)
Calcium ion binding (8)
Protein kinase activity (6)
Cytoskeletal protein binding (9)
Peptidase activity (9)
Enzyme regulatory activity (10)
Lipid binding (8)
Nuclease activity (5)
Nucleotide binding (28)
C
Cytoplasmic membrane-bounded vesicle (7)
Cytoskeleton (15)
Protein complex (50)
Cytosol (20)
Endoplasmic reticulum (5)
Extracellular space (7)
Plasma membrane (14)
Mitochondrium (14)
Nucleoplasm (25)
Nuclear chromosome (11)
Nuclear envelope (6)
Nucleolus (6)
