# Supplementary material for: Identification of conserved genes triggering puberty in European sea bass males (Dicentrarchus labrax) by microarray expression profiling
Source: BMC Genomics. 2017 Jun 5;18:441. doi: 10.1186/s12864-017-3823-2 (PMC5460432; doi:10.1186/s12864-017-3823-2)
Supplement: Supplementary file 6 — A table containing the microarray versus qPCR fold change (FC) expression values for 14 genes differentially expressed in the European sea bass transcriptome during early stages of pubertal development (word format, .doc). (DOC 54 kb) [file 12864_2017_3823_MOESM6_ESM.doc]

Additional file 6. Microarray versus qPCR fold change (FC) results for 14 genes in the sea bass transcriptome affected during early stages of pubertal development

|  |  | Microarray | | |  | qPCR | | |
| --- | --- | --- | --- | --- | --- | --- | --- | --- |
| Gene group | Gene symbol | Regulation* | FC | FC adjusted p-value |  | Regulation* | FC | FC p-value  (t-test) |
| Cell proliferation and cell cycle progression | *pcna* | up | 2.66 | 0.0014 |  | up | 4.43 | 0.016 |
|  | *cenpf* | up | 3.04 | 0.0011 |  | up | 3.91 | 0.043 |
|  | *spc25* | up | 3.60 | 0.0004 |  | up | 3.05 | 0.003 |
|  | *cenpi* | up | 2.12 | 0.0015 |  | no change | 0.51 | 0.339 |
|  | *trip13* | up | 3.51 | 0.0005 |  | up | 3.22 | 0.032 |
|  | *cdc28* | up | 5.16 | 0.0003 |  | up | 4.15 | 0.000 |
| Reproduction and growth | *amh* | down | 2.09 | 0.0041 |  | down | 3.07 | 0.007 |
|  | *sgII* | down | 2.10 | 0.0094 |  | down | 4.82 | 0.047 |
|  | *aqp1* | down | 2.29 | 0.0034 |  | down | 3.28 | 0.045 |
|  | *igfbp6* | up | 5.44 | 0.0089 |  | no change | 1.69 | 0.454 |
|  | *agrp2* | down | 2.65 | 0.0099 |  | down | 4.29 | 0.039 |
| RA signalling | *cyp26a1* | down | 2.97 | 0.0098 |  | down | 5.43 | 0.029 |
|  | *crabp1* | down | 2.62 | 0.0008 |  | down | 3.55 | 0.000 |
|  | *rbp4* | down | 3.37 | 0.0005 |  | down | 11.02 | 0.003 |

*Regulation = FC value in stage II compared with stage I (stage II/stage I).

Upregulated = up; downregulated = down
