# Supplementary material for: Identification of conserved genes triggering puberty in European sea bass males (Dicentrarchus labrax) by microarray expression profiling
Source: BMC Genomics. 2017 Jun 5;18:441. doi: 10.1186/s12864-017-3823-2 (PMC5460432; doi:10.1186/s12864-017-3823-2)
Supplement: Supplementary file 7 — A table containing all the protein sequences used to generate the phylogenetic tree for cyp26 (word format, .doc). (DOCX 16 kb) [file 12864_2017_3823_MOESM7_ESM.docx]

Additional file 7. CYP26 protein sequences used to infer the phylogenetic tree in figure 6

|  | Latin name | Common name | Protein symbol | GenBank/Ensembl accession number |
| --- | --- | --- | --- | --- |
| **Teleosts** | *Dicentrarchus labrax* | sea bass | Cyp26a1 | AHY95171 |
|  | *Gasterosteus aculeatus* | stickleback | Cyp26a1 | ENSGACP00000020277 |
|  |  |  | Cyp26b1 | ENSGACP00000024870 |
|  |  |  | Cyp26c1 | ENSGACP00000014662 |
|  | *Oreochromis niloticus* | tilapia | Cyp26a1 | ENSONIP00000009129 |
|  |  |  | Cyp26b1 | ENSONIP00000000575 |
|  |  |  | Cyp26c1 | ENSONIP00000019469 |
|  | *Xiphophorus maculatus* | platyfish | Cyp26a1 | ENSXMAP00000014170 |
|  |  |  | Cyp26b1 | ENSXMAP00000000560 |
|  |  |  | Cyp26c1 | ENSXMAP00000004904 |
|  | *Oryzias latipes* | medaka | Cyp26a1 | NP_001265772 |
|  |  |  | Cyp26b1 | ENSORLP00000004308 |
|  |  |  | Cyp26c1 | ENSORLT00000002541 |
|  | *Gadus morhua* | cod | Cyp26a1 | ENSGMOT00000014287 |
|  |  |  | Cyp26b1 | ENSGMOT00000005622 |
|  |  |  | Cyp26c1 | ENSGMOT00000001784 |
|  | *Tetraodon nigroviridis* | pufferfish | Cyp26a1 | ENSTNIP00000007321 |
|  |  |  | Cyp26b1 | ENSTNIP00000014173 |
|  |  |  | Cyp26c1 | ENSTNIP00000021864 |
|  | *Takifugu rubripes* | fugu | Cyp26a1 | ENSTRUP00000004613 |
|  |  |  | Cyp26b1 | ENSTRUP00000031925 |
|  |  |  | Cyp26c1 | ENSTRUP00000032385 |
|  | *Danio rerio* | zebrafish | Cyp26a1 | NP_571221 |
|  |  |  | Cyp26b1 | NP_997831 |
|  |  |  | Cyp26c1 | NP_001025122 |
|  | *Petromyzon marinus* | lamprey | Cyp26b1 | ENSPMAT00000000847 |
| **Amphibians** | *Xenopus laevis* | xenopus | Cyp26a1 | NP_001088938 |
|  |  |  | Cyp26b1 | ACF33501 |
|  |  |  | Cyp26c1 | NP_001089956 |
|  | *Silurana tropicalis* | silurana | Cyp26a1 | NP_001016147 |
|  |  |  | Cyp26b1 | ENSXETP00000046568 |
|  |  |  | Cyp26c1 | ENSXETP00000039433 |
| **Reptiles** | *Pelodiscus sinensis* | turtle | CYP26A1 | ENSPSIP00000020213 |
|  |  |  | CYP26B1 | ENSPSIP00000014920 |
|  |  |  | CYP26C1 | ENSPSIP00000020108 |
|  | *Anolis carolinensis* | lizard | CYP26A1 | ENSACAT00000006924 |
|  |  |  | CYP26C1 | ENSACAP00000006744 |
| **Birds** | *Meleagris gallopavo* | turkey | CYP26A1 | ENSMGAP00000009264 |
|  |  |  | CYP26B1 | ENSMGAP00000014077 |
|  | *Gallus gallus* | chicken | CYP26A1 | NP_001001129 |
|  |  |  | CYP26B1 | XP_015141554 |
|  |  |  | CYP26C1 | XP_421678 |
|  | *Anas platyrhynchos* | duck | CYP26A1 | ENSAPLP00000002315 |
|  |  |  | CYP26B1 | ENSAPLP00000007277 |
|  |  |  | CYP26C1 | ENSAPLP00000002568 |
|  | *Ficedula albicollis* | flycatcher | CYP26A1 | ENSFALP00000008250 |
|  |  |  | CYP26B1 | ENSFALP00000011370 |
|  |  |  | CYP26C1 | ENSFALP00000008251 |
|  | *Taeniopygia guttata* | zebrafinch | CYP26A1 | ENSTGUP00000009117 |
|  |  |  | CYP26C1 | ENSTGUP00000009124 |
| **Mammals** | *Mus musculus* | mouse | CYP26A1 | NP_031837 |
|  |  |  | CYP26B1 | NP_780684 |
|  |  |  | CYP26C1 | NP_001098671 |
|  | *Pan troglodytes* | chimpanzee | CYP26A1 | ENSPTRP00000044339 |
|  |  |  | CYP26B1 | ENSPTRP00000020680 |
|  |  |  | CYP26C1 | ENSPTRP00000004819 |
|  | *Homo sapiens* | human | CYP26A1 | NP_000774 |
|  |  |  | CYP26B1 | NP_063938 |
|  |  |  | CYP26C1 | NP_899230 |
